# N-doped catalytic graphitized hard carbon for high-performance lithium/sodium-ion batteries

**DOI:** 10.1038/s41598-018-28310-3

**Published:** 2018-07-02

**Authors:** Ning Wang, Qinglei Liu, Boya Sun, Jiajun Gu, Boxuan Yu, Wang Zhang, Di Zhang

**Affiliations:** 1 State Key Laboratory of Metal Matrix Composites, School of Materials Science and Engineering, Shanghai Jiao Tong University, 800 Dongchuan Road, Shanghai, 200240 P.R. China; 2 CRRC Industrial Institute Co., Ltd, Beijing, China

## Abstract

Hard carbon attracts wide attentions as the anode for high-energy rechargeable batteries due to its low cost and high theoretical capacities. However, the intrinsically disordered microstructure gives it poor electrical conductivity and unsatisfactory rate performance. Here we report a facile synthesis of N-doped graphitized hard carbon via a simple carbonization and activation of a urea-soaked self-crosslinked Co-alginate for the high-performance anode of lithium/sodium-ion batteries. Owing to the catalytic graphitization of Co and the introduction of nitrogen-functional groups, the hard carbon shows structural merits of ordered expanded graphitic layers, hierarchical porous channels, and large surface area. Applying in the anode of lithium/sodium-ion batteries, the large surface area and the existence of nitrogen functional groups can improve the specific capacity by surface adsorption and faradic reaction, while the hierarchical porous channels and expanded graphitic layers can provide facilitate pathways for electrolyte and improve the rate performance. In this way, our hard carbon provides its feasibility to serve as an advanced anode material for high-energy rechargeable lithium/sodium-ion batteries.

## Introduction

In the past two decades, lithium-ion batteries (LIBs) have occupied the main market of energy storage devices owing to their light weight, high energy density and long cycle life^1–5^. However, with the explosive growing market of portable electronics and electrical vehicles, the scarcity and maldistribution of lithium resources make the future of LIBs full of challenges. Sodium-ion batteries (SIBs), as an alternative for LIBs, have attracted much attention recently, due to the low cost and earth abundance of sodium resources^6–14^. Being in the same main group, sodium owns similar electrochemical properties compared to lithium, only with its radius a little larger than lithium (1.02 nm for Na^+^ vs. 0.76 nm for Li^+^)^15^. Therefore, it is meaningful to develop advanced electrode materials available for both LIBs and SIBs.

Graphitic carbon, now commonly used as the anode of commercial LIBs, using its long range ordered graphitic layers to store lithium (theoretical capacity 372 mAh g^−1^), cannot supply enough energy for electric vehicles^10,16,17^. One proposed solution is hard carbon, usually synthesized by pyrolysis of polymers. Hard carbon consists mainly of single graphene layers randomly packed in a disordered arrangement^18–24^. This structure usually provides not only broad parallel carbon layers but also numerous nanopores for Li^+^/Na^+^ intercalation, thus giving larger specific capacities^25–27^. However, the highly disordered structure of hard carbon bear defects of low conductivity and poor electrochemical stability, which lead to poor rate performance and cyclic stability^10^. Pervious works mainly focused on introducing a graphitic component^16,28^ or creating a more ordered graphene morphology^29,30^, but the product still retained its disordered structure.

In this work, we present a facile solution to improve the electrochemical performance of hard carbon by an in-suit catalytic graphitization and N-doping method. By simple carbonization and activation of a urea soaked self-crosslinked catalytical metal-alginate, we successfully synthesized a N-doped graphitized hard carbon (N-GHC). The obtained N-GHC owns merits of ordered broad interlayer distance (0.36 nm), hierarchical porous channels, large surface area (1008 m^2^ g^−1^), as well as abundant nitrogen and oxygen functional groups. When applied in the anode of LIBs and SIBs, the large surface area and abundant nitrogen and oxygen functional groups can help generate high lithium and sodium storage capacity through surface adsorption and faradic reaction^31–36^, while the expanded graphitic layers and hierarchical porous channels can accelerate the transportation of electrolyte and improve the rate performance.

## Results and Discussion

### Microstructure of the N-GHC

The N-GHC was synthesized by carbonization and activation of a urea-soaked catalytical metal-alginate precursor. The natural polymer alginate can self-crosslink with multivalent metal ions including the catalytical metal ions like Co^2+^, Ni^2+^, Fe^3+^, etc. to form the uniform metal-alginate gels^37^. Among the various catalytical metal ions, the divalent Co^2+^ with strong crosslinking nature with alginate and relatively high catalytical graphitization effect was chosen as a represent. Briefly, sodium alginate solutions were added into a Co(NO_3_)_2_ solution to crosslink with Co^2+^ and make the Co-alginate. Then the Co-alginate was freeze-dried and immersed into a urea solution. After drying and carbonization, a N-Co/C composite was obtained. Then the N-Co/C was activated by KOH and washed with HCl to remove the Co catalyst and get the final N-GHC.

**Figure 1:**
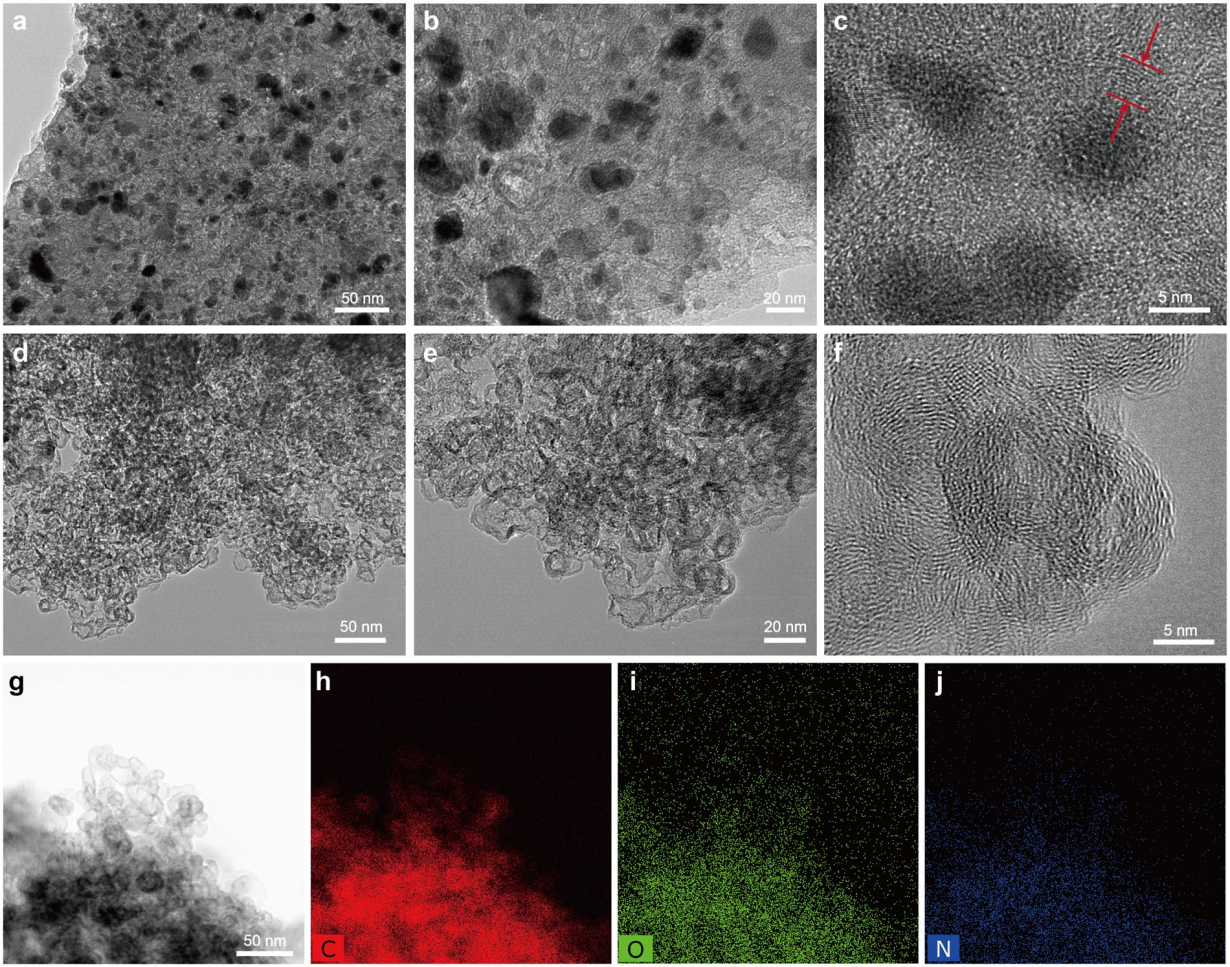
TEM images of the N-Co/C (**a**–**c**) and N-GHC (**d**–**f**). STEM image (**g**) and corresponding C- (**h**), O- (**i**) and N- (**j**) elemental mapping of the N-GHC.

Figure 1 shows the TEM images of the as-prepared N-Co/C and N-GHC samples. During carbonization, the Co^2+^ in the urea-Co-alginate turned into dense Co particles with diameter of 5–10 nm, while the organic chains turned into carbon matrix. Due to the catalytic graphitization of Co, the carbon texture near Co particles was rearranged into ordered graphitic layers^38–40^, as seen in Fig. 1a–c. These Co particles were tightly protected by the ordered graphitic layers and cannot be removed by simply acid washing (Figs S1 and S2). To remove the Co particles and get the N-GHC, an activation of KOH and further acid washing of HCl were employed. The results shown that all the Co particles were moved away, only leaving the hollow graphitized carbon layers, as seen in Figs 1d–f and S3. These graphitized carbon layers are about 5–10 layers in thickness, and the interlayer distance is 0.36 nm. Unlike the smooth graphitic layers in graphite, the graphitized carbon layers in our sample are wrinkled due to the activation of KOH. KOH activation also broaden the interlayer distance and bring in porous channels in between the graphitic layers^34^, which may facilitate Li^+^/Na^+^ diffusion and storage. Furthermore, the bright-field scanning transmission electron microscopy (STEM) image (Fig. 1g) and corresponding elemental mapping (Fig. 1h–j) of the N-GHC show that oxygen and nitrogen heteroatoms are uniformly distributed in the carbon material. The existence of these heteroatoms may contribute to the Li^+^/Na^+^ storage capacity through faradic reactions.

**Figure 2:**
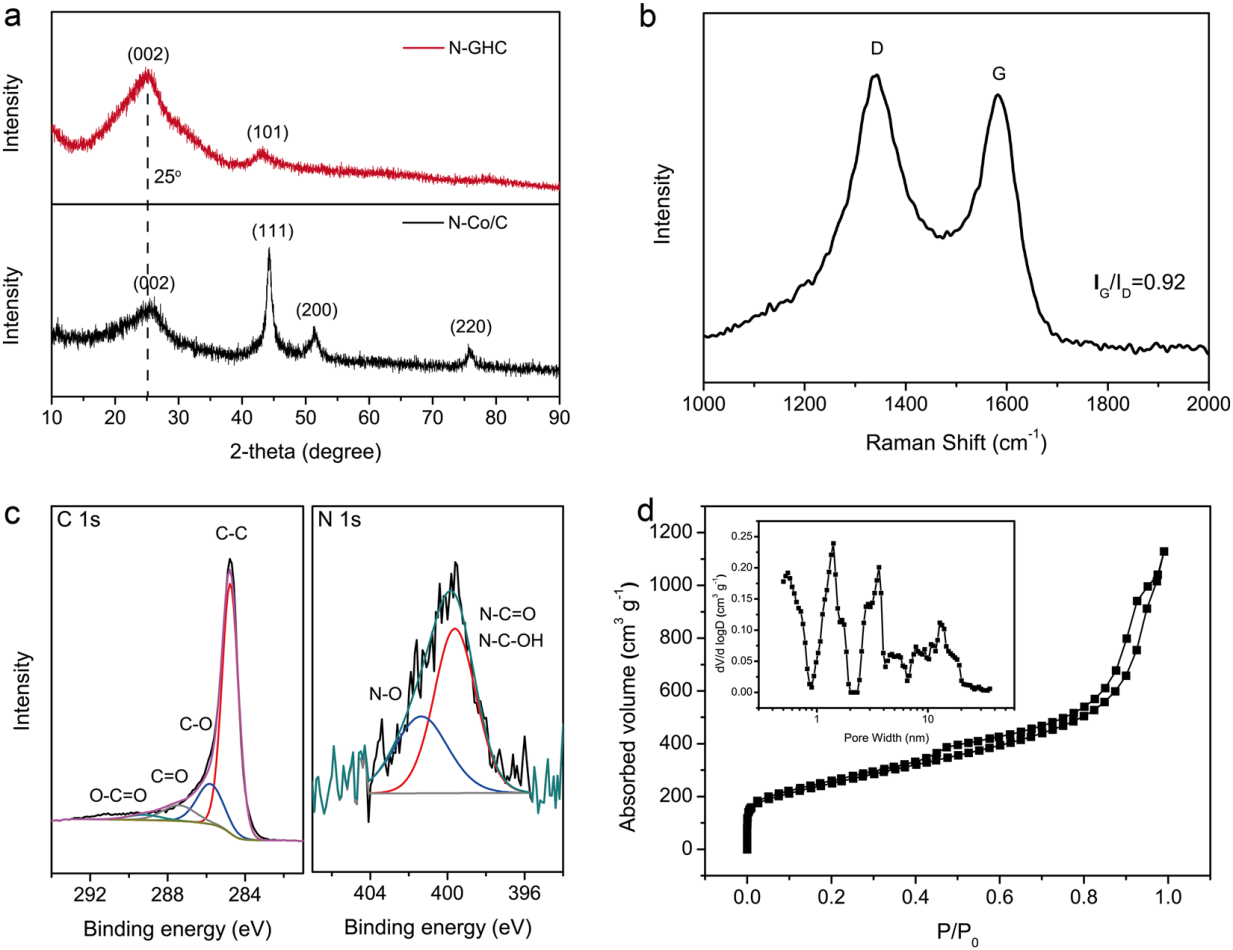
Characterizations of the N-GHC. (**a**) XRD patterns. (**b**) Raman spectrum. (**c**) High-resolution XPS spectra of the C1s and N1s peak. (**d**) N_2_ sorption isotherm and pore size distribution.

Figure 2a shows the XRD patterns of the N-Co/C and N-GHC. For N-Co/C sample, the sharp peaks at 44.2, 51.5, and 75.8° are ascribed to the diffraction of metallic Co, while the peak at 25° corresponds to the (002) diffraction of the graphitic layer-to-layer structure. As for N-GHC, the peaks for metallic Co disappeared, only leaving two peaks at 25° and 44°, corresponding to the (002) and (101) plane of graphite. Based on the Bragg equation (2dsinθ = λ) and the position of (002) peak, the interlayer distance of graphitic carbons is calculated to be 0.36 nm^24^, which is in accordance with the HR-TEM (Fig. 1f). The Raman spectrum of N-GHC in Fig. 2b indicates the coexistence of ordered and disordered carbons. The Raman peak at 1583 cm^−1^ (G-band) corresponds to the sp^2^ orbital structure of the ordered graphitic layers, while the peak at 1343 cm^−1^ (D-band) represents the lattice defects and disordered carbons^41,42^. The *I*_*G*_*/I*_*D*_ value is calculated to be 0.92, representing a relatively high degree of graphitization. XPS measurement demonstrates that the weight percentage of C, O, and N in our N-GHC are 94.33%, 4.35% and 1.32%, respectively. Figure 2c shows the high resolution XPS spectra of C1s and N1s. The C1s peak is fitted into four peaks centered at 284.8, 286.0, 287.5, and 289.2 eV, corresponding to the C-C, C-O, C=O, and O-C=O bonds, respectively. The N1s peak of N-HPC is composed of pyridinic N (399.9 eV) and N-oxides (401.5 eV). The existence of these O- and N- functional groups can add the wettability of the electrode and provide extra faradic capacity^43,44^. N_2_ adsorption-desorption measurement indicates that the N-GHC owns a hierarchical porous structure (Fig. 2d). In the isotherm, the N_2_ adsorption in low relative pressure is ascribed to micropores, the big hysteretic loop in middle relative pressure corresponds to the capillary condensation of mesopores, while the N_2_ adsorption over high relative pressure represents the existence of macropores. The Brunauer-Emmett-Teller (BET) specific surface area is calculated to be 1008 m^2^ g^−1^. The corresponding pore size distribution inserted in Fig. 2d exhibits abundant micropores below 2 nm, and large numbers of mesopores at 2–50 nm. These small mesopores and micropores were produced by metallic Co removal and KOH activation. Such hierarchical porous structure can provide fast transportation channels for electrolyte ions to access into the graphitic carbon layers, and give extra capacity through surface adsorption^43,45^.

For comparison, the N-GHC catalyzed by Ni, Fe and Cu were also synthesized by the same method, donating as N-GHC-Ni/Fe/Cu (Figs S4–S10). The N-GHC-Ni also shows mesoporous and highly graphitized nanostructure like N-GHC-Co (Fig. S4). This is mainly because of the close crosslink ability and catalytic graphitization ability of Ni and Co. The N-GHC-Fe exhibits microporous structure with lower degree of graphitization as compared to the N-GHC-Co (Fig. S6). This should be attributed to the weaker catalytical graphitization of Fe_3_O_4_. As for the Cu catalyst, the N-Cu/C hybrid shows relatively high degree of graphitization, since there are thick graphitic carbon layers around the Cu particles in the TEM images (Fig. S8) and obvious (002) peak for graphitic carbons in the XRD pattern (Fig. S9). However, after activation with KOH and acid washing with HCl, the Cu catalyst turns into complex mixture of metallic Cu, Cu_2_O, CuCl and other Cu-containing chemicals, which cannot be easily removed. Therefore, using Cu as catalyst to make the N-doped graphitized hard carbon in this method remains to be modified for the removal of Cu catalyst.

### Electrochemical performance of N-GHC in LIBs

**Figure 3:**
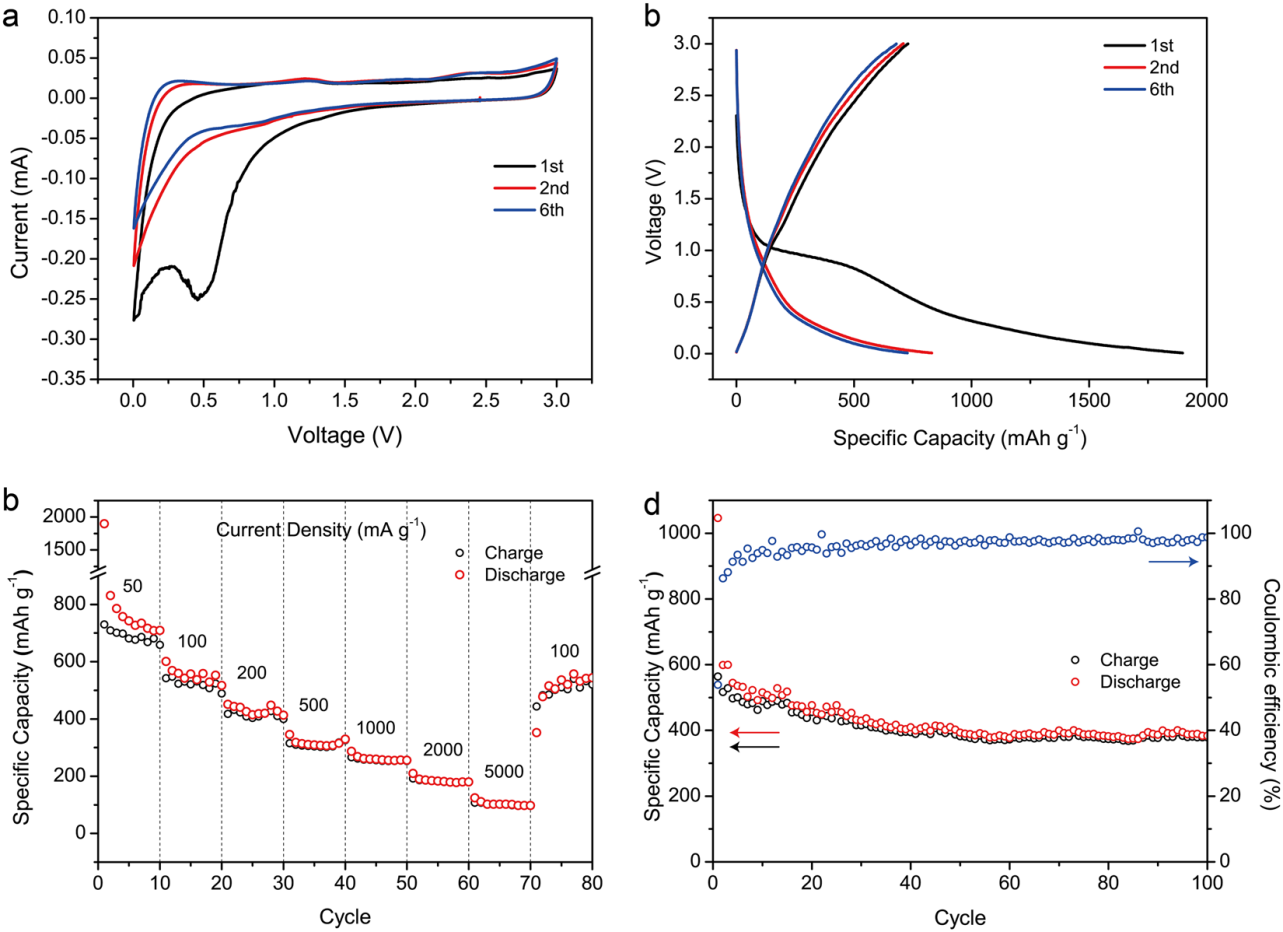
Electrochemical performance of N-GHC in LIBs. (**a**) First two and sixth CV curves at 0.1 mV s^−1^. (**b**) First two and sixth GCD profiles at 100 mA g^−1^. (**c**) Rate performance at various current densities. (**d**) Cycling performance at 100 mA g^−1^.

The N-GHC catalyzed by Co was chosen as a represent to show the electrochemical performance. For the lithium storage performance test, the N-GHC was assembled into Li half-cells and measured in the voltage window of 0.005–3 V. Figure 3a shows the first two and sixth CV curves at scan rates of 0.1 mV s^−1^. In the first cathodic process, the sharp peak near 0.5 V corresponds to the irreversible decomposition of electrolyte and formation of solid electrolyte interphase (SEI) film^29,46^, while the higher peak below 0.1 V represents Li^+^ intercalation into the broad graphitic layers in the N-GHC. In the following cycles, the cathodic peak at 0.5 V disappeared, and the sharp Li^+^ intercalation peak below 0.1 V remains. In the anodic process, the broad peak near 0.2 V corresponds to Li^+^ extraction out of the graphitic carbon layers, which is the inverse process of Li^+^ intercalation. In addition to the Li^+^ intercalation and extraction peaks below 0.2 V, the near rectangular CV shape over 0.2–3 V is attributed to the surface adsorption of Li^+^ by micropores, as well as the surface faradic reactions between Li^+^ and N-, O-functional groups. Figure 3b shows the first two and sixth GCD profiles at current density of 50 mA g^−1^. In the discharge curves, the plateau below 0.1 V corresponds to Li^+^ intercalation into graphitic layers, while the slope above 0.1 V represents Li^+^ storage by surface adsorption and faradic reactions, which are in good accordance with the CV curves.

Figure 3c shows the rate performance of N-GHC with current densities ranging from 50 to 5000 mA g^−1^. In the first cycle at 50 mA g^−1^, a relatively high discharge capacity of 1797 mAh g^−1^ is obtained and it quickly drops to 831 mAh g^−1^ in the second cycle. This is mainly caused by the irreversible electrolyte decomposition and SEI formation in the first cycle. In the latter cycles, the specific capacity slightly decreases to 710 mAh g^−1^ in the tenth cycle. The specific capacity of the N-GHC at 100, 200, 500, and 1000 mA g^−1^ are calculated to be 517, 413, 329, and 256 mAh g^−1^, respectively. Even at a high current density of 2000 mAh g^−1^, it still delivers capacity of 180 mAh g^−1^. The specific capacity of our N-GHC is higher than most reported literatures, like graphene nanosheets (GNS)^47^, CNT^48^, GNS/CNT^49^, GNS/C_60_^49^, potato derived hard carbons^50^ and pitch modified hard carbon^51^, as listed in Table S1. The high specific capacity and good rate performance is ascribed to the hierarchical porous channels, broad interlayer distance, and abundant surface functional groups. The hierarchical porous channels can facilitate the transportation of electrolyte ions at high current densities and provide abundant surface area for the adsorption of Li^+^. The broadened interlayer distance of our N-GHC can accelerate the Li^+^ intercalation in between the graphitic layers and improve the specific capacity. The abundant surface functional groups can store lithium through faradic reactions. Thus, high specific capacity and good rate performance are obtained. Figure 3d shows the cycling performance at 100 mA g^−1^. After 100 cycles, the capacity remains high value of 389 mAh g^−1^, which is 77% of the tenth cycle (507 mAh g^−1^). This excellent cycling stability of our N-GHC is mainly ascribed to its rational porous transportation channels and physical robust nanostructure.

### Electrochemical performance of N-GHC in SIBs

**Figure 4:**
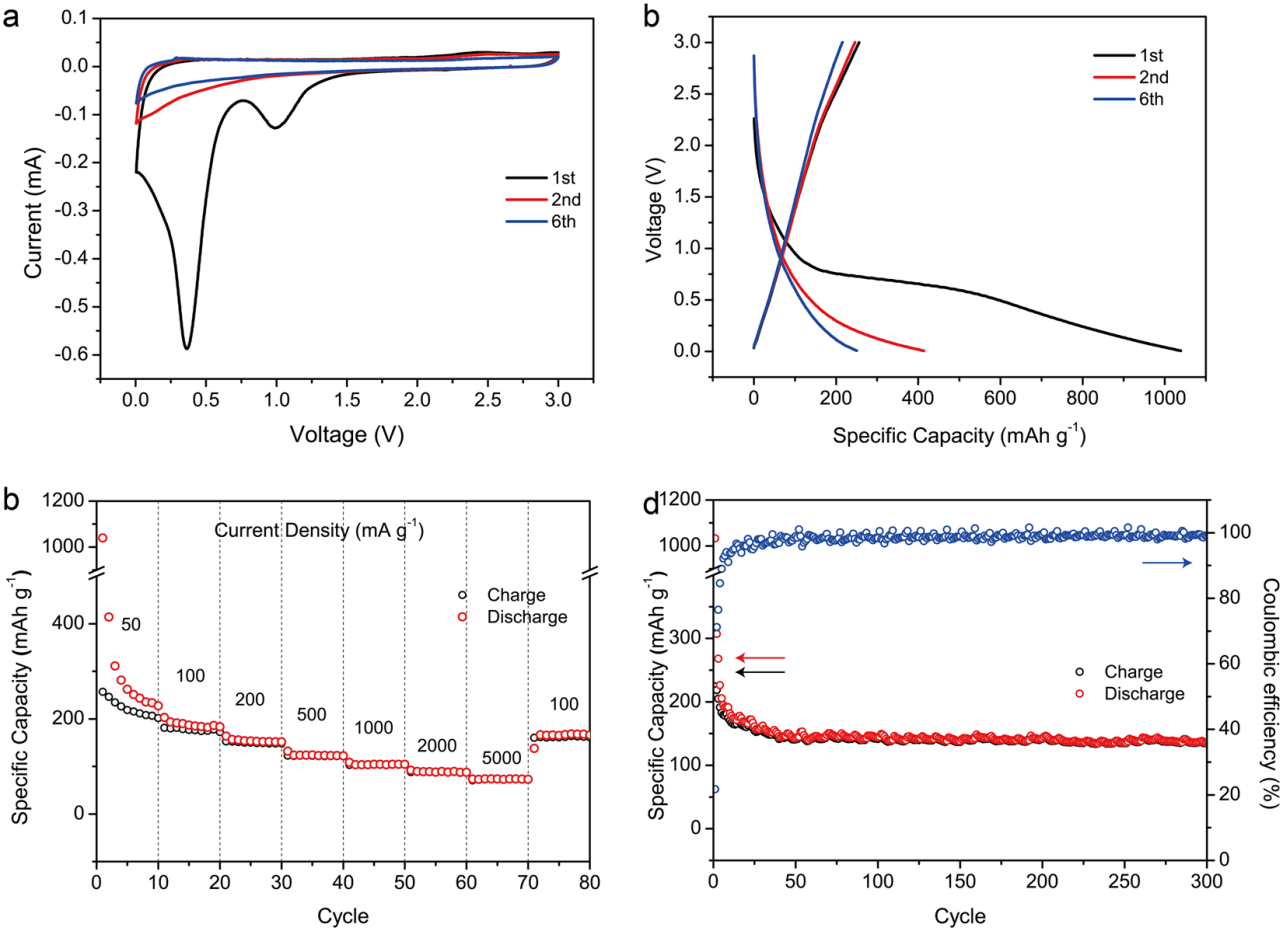
Electrochemical performance of N-GHC in SIBs. (**a**) First two and sixth CV curves at 0.1 mV s^−1^. (**b**) First two and sixth GCD profiles at 50 mA g^−1^. (**c**) Rate performance. (**d**) Cycling performance at 100 mA g^−1^.

The sodium storage performance of N-GHC was tested in sodium half-cells within voltage window of 0.005–3 V. Figure 4a shows the first two and sixth CV curves at scan rate of 0.1 mV s^−1^. In the first cycle, the two cathodic peaks at 1.0 and 0.4 V are ascribed to the electrolyte decomposition and SEI formation^7,8,52^. In the following cycles, the sharp cathodic peaks at 0.1 V and broad anodic peaks near 0.2 V correspond to the Na^+^ intercalation/extraction into/out of the broad graphitic carbon layers, which are in similar with those in LIBs. Compared to the CV curves for N-GHC in LIBs, the CV peaks in SIBs are broader and weaker. This is related to the larger radius of Na^+^ than Li^+^ and the more sluggish Na^+^ intercalation in between the graphitic carbon layers^17,53,54^. The GCD profiles in Fig. 4b shows smooth slopes without apparent plateaus, which matches well with the broad CV peaks.

Figure 4c exhibits the rate performance of N-GHC at various current densities. The specific capacity in the first cycle is 1039 mAh g^−1^ and it quickly drops to 414 mAh g^−1^ in the second cycle. This is mainly caused by the irreversible electrolyte decomposition and SEI film formation in the first cycle, the same with the situation in LIBs. In the tenth cycle, a lower but stable specific capacity of 227 mAh g^−1^ is obtained. The specific capacity of the N-GHC in the anode of SIBs at 100, 200, 500, and 1000 mA g^−1^ are calculated to be 186, 152, 122, and 104 mAh g^−1^, respectively. After 70 cycles at various current densities, a high specific capacity of 166 mAh g^−1^ is still recovered in the 71th cycle, as current density turns back to 100 mA g^−1^. The specific capacity of our N-GHC in SIBs is higher than natural graphite^55^, N-carbon nanofibers^56^, and is comparable to other reported literatures, like expanded graphite^57^, ion-catalyzed hard carbon^10^, carbon nanofibers^58^, defective graphene^59^ etc. as listed in Table S2. The reasonable specific capacities and good rate performance of our N-GHC in SIBs are also attributed to the hierarchical porous channels, broad interlayer distance and abundant surface functional groups. Figure 4d shows the cycling performance in 100 mA g^−1^. After 300 cycles, the specific capacity remains 136 mAh g^−1^, which is 76% of the tenth capacity (178 mAh g^−1^), indicating excellent cycling stability.

**Figure 5:**
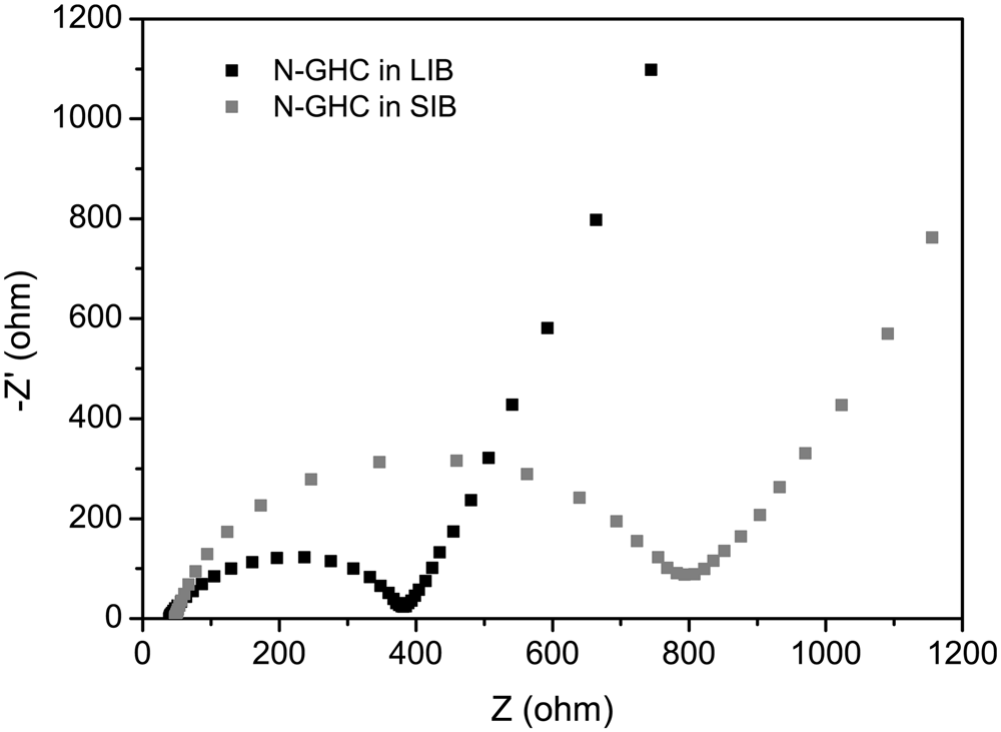
The Nyquist plots of the N-GHC electrode in LIBs and SIBs.

Despite of the reasonable specific capacities and good rate performance compared with previous works, the specific capacities of N-GHC in SIBs are still much lower than in LIBs in the same current density. Various dual-role anode materials have been reported with lower capacity in SIBs than in LIBs^10,60^, but the reason for this remains unclear. To better study the sodium storage and lithium storage processes in the N-GHC based electrode, EIS data were measured after six CV cycles in LIBs and SIBs. As shown in the Nyquist plots in Fig. 5, the intersections in the X-axis at high frequency region represent the electronic connection resistance of cells (R_s_), which are very close for N-GHC in LIBs and SIBs. The diameter of the semicircles at medium frequency region stand for the charge-transfer resistance (R_ct_) in the interfaces of electrode and electrolyte. The much lower R_ct_ for N-GHC in LIBs indicates that the Li^+^ transfer from electrolyte to the surface of electrode is much easier than Na^+^. The slope of the Nyquist plots at low frequency region corresponds to the diffusive resistance (Z_w_, Warburg impedance) of electrolyte ions inside the electrode. The higher slope for N-GHC in LIBs indicates that Li^+^ bear less resistance during diffusing inside the N-GHC electrode as compared to Na^+^. Through the EIS analysis, we have found out that compared to Li^+^, Na^+^ intercalation in and extraction out from the N-GHC electrode suffers from larger charge-transfer impedance and diffusion impedance. This may lead to worse reactive kinetics and lower capacities of N-GHC in SIBs than in LIBs.

## Conclusions

In conclusion, we have demonstrated a facile synthesis of N-GHC through simple carbonization and activation of urea-soaked self-crosslinked Co-alginate. Owing to the catalytic graphitization of Co, activation of KOH, and nitrogen functional groups introduction of urea, the obtained N-GHC owns merits of ordered expanded graphitic layer structure, hierarchical porous channels, large surface area, and abundant functional groups. Applying in the anode of LIBs and SIBs, the large surface area and the existence of nitrogen functional groups can improve the specific capacity through surface adsorption and faradic reaction, while the hierarchical porous channels and expanded graphitic layers can provide facilitate pathways for electrolyte and improve the rate performance. In this way, our hard carbon provides its feasibility to serve as an advanced anode material for high-energy rechargeable lithium/sodium-ion batteries.

## Methods

### Material synthesis

Sodium alginate was dissolved into deionized water to form a 1.5 wt.% aqueous solution. Then 400 mL of the sodium alginate solution was added into 500 mL of 5 wt.% cobalt nitrate solution to obtain the Co-alginate hydrogel. Then the Co-alginate hydrogel was frozen by liquid nitrogen and dried in a freeze-dryer for 24 h. The dried Co-alginate was soaked into a urea solution for 12 h under a weight ratio of m_Co-alginate_:m_urea_ = 1:2. The obtained urea-Co-alginate was dried in a blast drier, and then carbonized under N_2_ atmosphere at 600 °C for 1 h to obtain N-doped Co/C sample. To remove the metallic Co particles and bring in pores, the N-doped Co/C samples were first mixed with KOH with weight ratio of 1:4, and then heated under 750 °C for 1 h in N_2_ atmosphere. The obtained sample was washed with hydrochloric acid to remove Co particles and alkaline substances, and then washed with deionized water to obtain to final N-GHC. The N-GHC-Ni, -Fe and -Cu were synthesized by the same way as N-GHC-Co, only changing the solution of Co(NO_3_)_2_ to Ni(NO_3_)_2_, FeCl_3_ and CuCl_2_.

### Material characterization

Microstructures of the obtained N-metal/C hybrids and N-GHCs were observed by a scanning electron microscope (SEM, FEI Quanta FEM 250) and a transmission electron microscope (TEM, JEOL JEM-2100F). Chemical compositions of the obtained samples were analyzed by X-ray diffraction (XRD, Rigaku D/Max-2550, Cu-Kα radiation), Raman spectrum (Renishaw Via-reflex spectrometer), and X-ray photoelectron spectroscopy (XPS, PHI 5700 ESCA). Pore characteristics of the N-GHC were studied by nitrogen adsorption-desorption isotherm on a Quantachrome Autosorb-iQ at 77 K. The corresponding pore size distribution of the N-GHC was calculated by the density function theory (DFT) method.

### Electrochemical measurements

Electrochemical performance of the N-GHC was measured in the Li-half cells and Na-half cells. For the electrode preparation, active material (N-GHC) was mixed with Super P (conductive additive) and poly-(vinyl difluoride) (PVDF, binder) under the weight ratio of 8:1:1 in the solution of N-methyl-pyrrolidone (NMP) and stirred for 24 h to make a uniform slurry. Then the mixture slurry was coated on a copper foil with coating thickness of 100 μm. The coated copper foils were then dried at 110 °C for 12 h in vacuum, and then cut into disks with diameter of 11 mm. These disks were latter pressed at 5 MPa for 1 minute and dried for another 12 h. The average loading of active materials is 0.8~1 mg cm^−2^. The dried electrodes were transferred to Ar glove box for battery assembling. For Li-half cells, the counter electrode was lithium foil, the separator was the Celgard 2500, and the electrolyte was a 1 M LiPF_6_ in mixture of dimethyl carbonate (DMC) and ethylene carbonate (EC) (v/v = 1:1). As for Na-half cells, the counter electrode was sodium foil, the separator was the glass fibers from Whatman (GF/D), while a 1 M NaClO_4_ in mixture of ethylene carbonate (EC) and propylene carbonate (PC) (v/v = 1:1) was employed as the electrolyte. The electrochemical performance of the Li-half cells and Na-half cells was evaluated through Galvanostatic charge-discharge (GCD) measurement, cyclic voltammetry (CV) measurement and electrochemical impedance spectroscopy (EIS) plots. The GCD were conducted on a Land CT2001A (China), while the CV and EIS were measured using a VMP3 electrochemical working station (France).

## Electronic supplementary material

Supplementary Information
