# Supplementary material for: N-doped catalytic graphitized hard carbon for high-performance lithium/sodium-ion batteries
Source: Sci Rep. 2018 Jul 2;8:9934. doi: 10.1038/s41598-018-28310-3 (PMC6028452; doi:10.1038/s41598-018-28310-3)
Supplement: Supplementary file 1 — Supplementary Information [file 41598_2018_28310_MOESM1_ESM.docx]

**N-doped catalytic graphitized hard carbon for high-performance lithium/sodium-ion batteries**

*Ning Wang^1^, Qinglei Liu*^1^, Boya Sun^1^, Jiajun Gu^1^, Boxuan Yu^2^, Wang Zhang^1^ and Di Zhang^1^*

^1^State Key Laboratory of Metal Matrix Composites, Shanghai Jiao Tong University, 800 Dongchuan Road, Shanghai, 200240, P.R. China

^2^CRRC Industrial Institute Co., Ltd

Corresponding author. Tel.: Fax: +86 21 34202634.

E-mail address: liuqinglei@sjtu.edu.cn (Q.L. Liu)





**Figure S1** TEM image of the N-Co/C after acid washing by HCl for 12h.


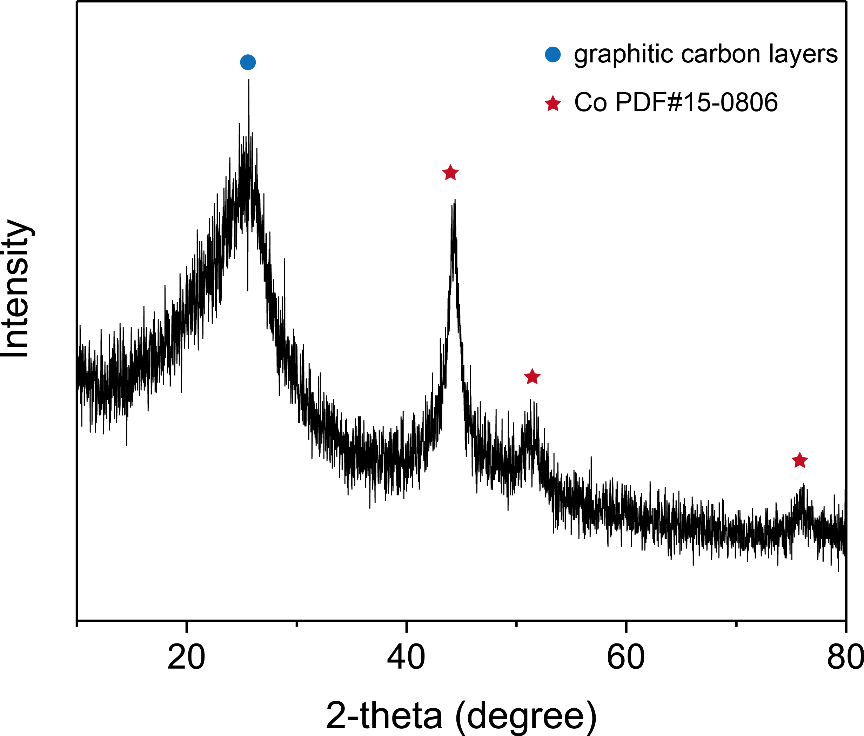


**Figure S2** XRD pattern of the N-Co/C after acid washing by HCl for 12h.


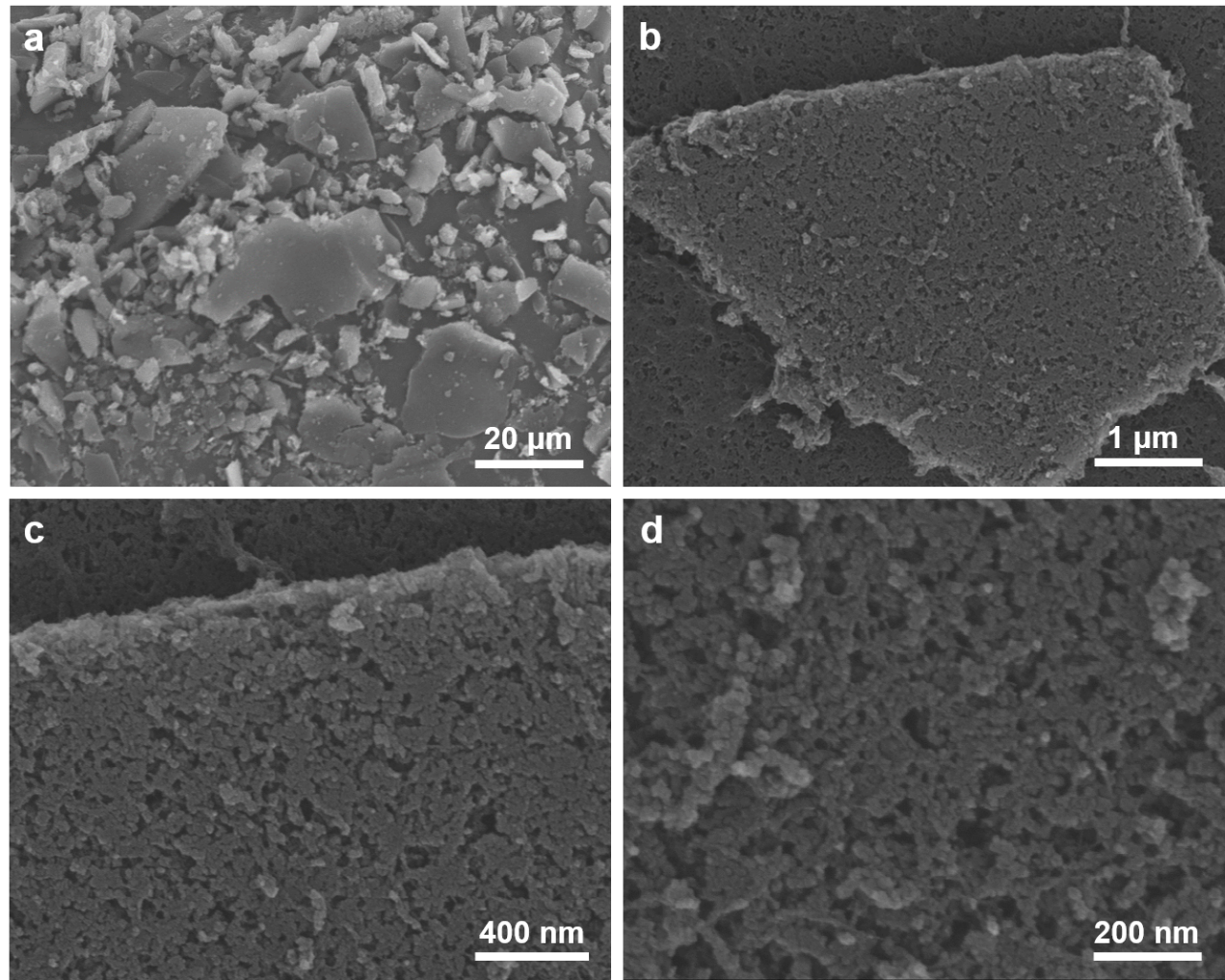


**Figure S3** SEM images of the N-GHC.


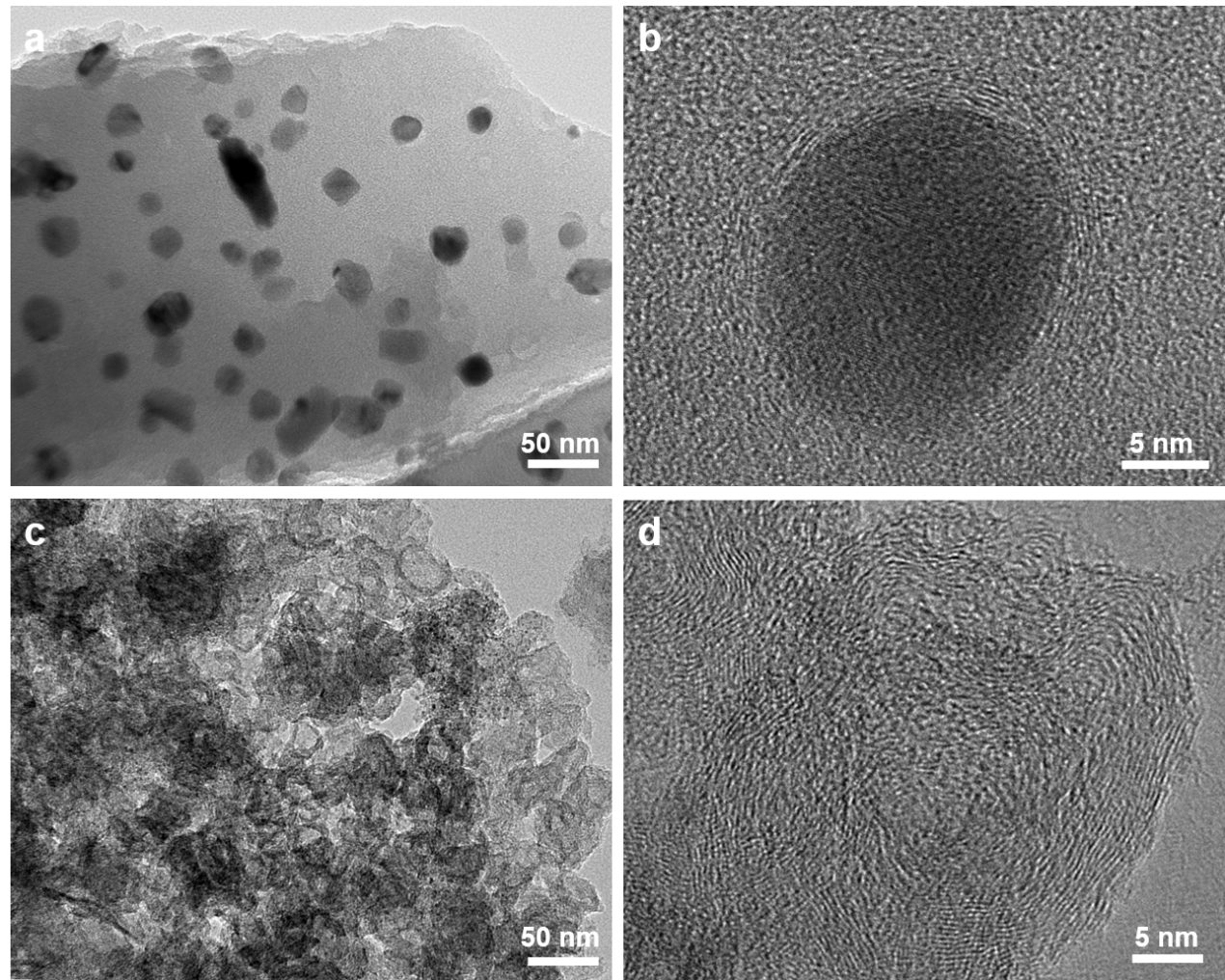


**Figure S4** TEM images of N-Ni/C (**a, b**) and N-GHC-Ni (**c, d**).

**Figure S5** XRD patterns of the N-Ni/C and N-GHC-Ni.


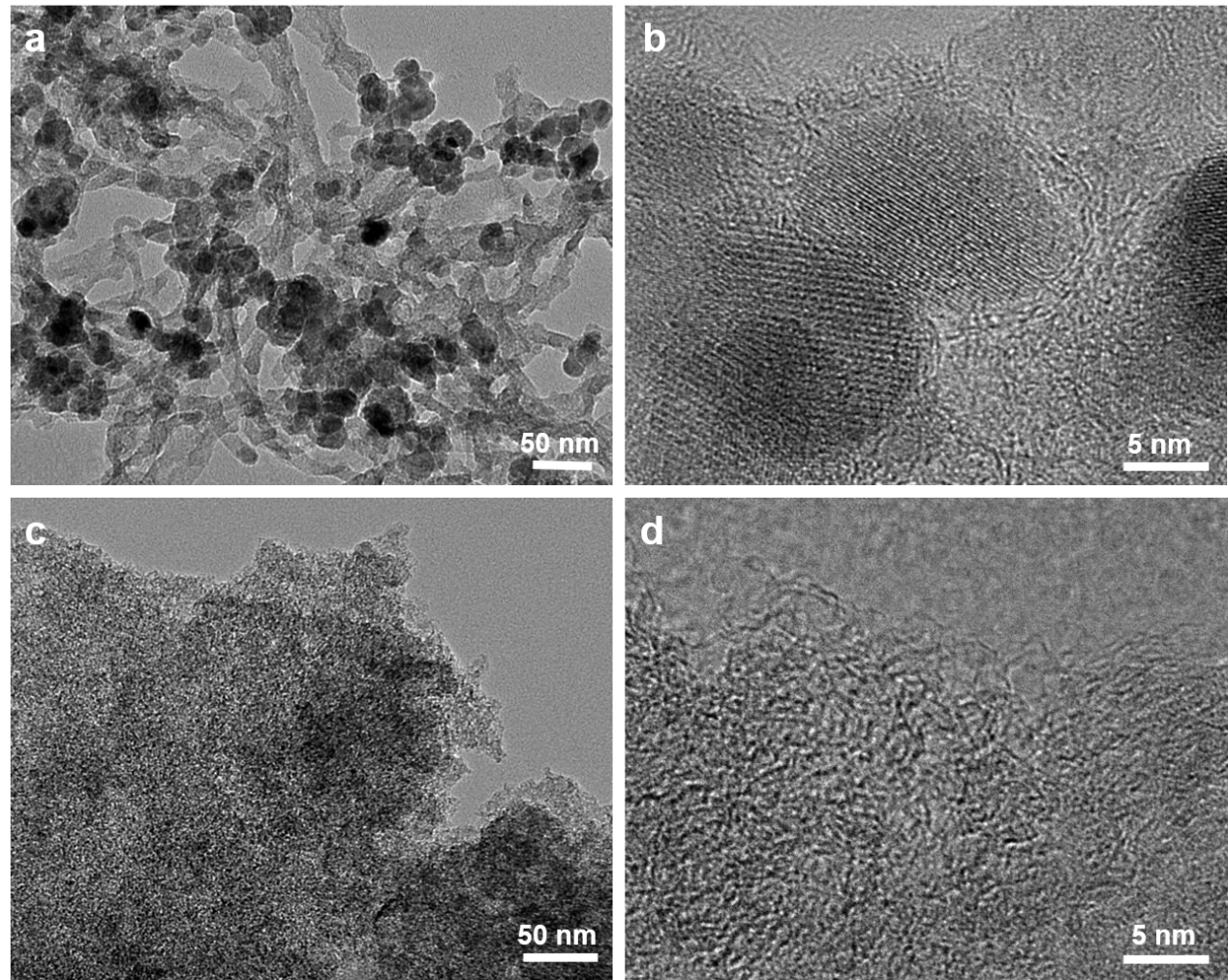


**Figure S6** TEM images of N-Fe_3_O_4_/C (**a, b**) and N-GHC-Fe (**c, d**).

**Figure S7** XRD patterns of the N-Fe_3_O_4_/C and N-GHC-Fe.


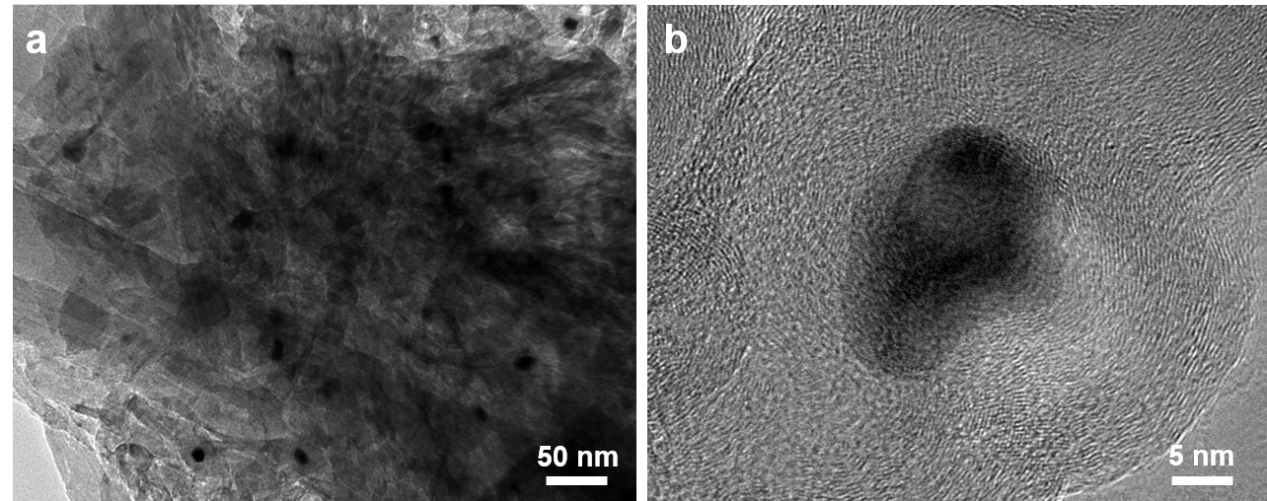


**Figure S8** TEM images of N-Cu/C.

**Figure S9** XRD pattern of the N-Cu/C.

**Figure S10** XRD pattern of the N-Cu/C after KOH activation and HCl washing.

**Table S1** Electrochemical performance of carbon materials in the anode of LIBs.

| Carbon material | Low current capacity | High current capacity | Reference |
| --- | --- | --- | --- |
| N-GHC | 50 mA g^-1^ 710 mAh g^-1^ | 1000 mA g^-1^ 256 mAh g^-1^ | This work |
| Graphene nanosheets (GNS) | 372 mA g^-1^ 460 mAh g^-1^ | - | ^1^ |
| GNS+C_60_ | 50 mA g^-1^ 600 mAh g^-1^ | - | ^2^ |
| CNF/GNS | 0.12 mA cm^-2^ 667 mAh g^-1^ | 6 mA cm^-2^ 189 mAh g^-1^ | ^3^ |
| N-graphene | 50 mA g^-1^ 872 mAh g^-1^ | 5000 mA g^-1^ 296 mAh g^-1^ | ^4^ |
| CNT | 200 mA g^-1^ 240 mAh g^-1^ | 400 mA g^-1^ 60 mAh g^-1^ | ^5^ |
| CNT/mesoporous carbon | 744 mA g^-1^ 380 mAh g^-1^ | 3720 mA g^-1^ 180 mAh g^-1^ | ^6^ |
| Hollow graphitic carbon nanosheets (HGCNS) | 100 mA g^-1^ 722 mAh g^-1^ | 3720 mA g^-1^ 380 mAh g^-1^ | ^7^ |
| CNT/HGCNS | 50 mA g^-1^ 960 mAh g^-1^ | 3700 mA g^-1^ 330 mAh g^-1^ | ^8^ |
| Potato derived hard carbon | 37 mA g^-1^ 531 mAh g^-1^ | 1860 mA g^-1^ 450 mAh g^-1^ | ^9^ |
| Pitch modified hard carbon | 186 mA g^-1^ 290 mAh g^-1^ | 3720 mA g^-1^ 160 mAh g^-1^ | ^10^ |
| Hollow carbon nanospheres | 37 mA g^-1^ 630 mAh g^-1^ | 1860 mA g^-1^ 229 mAh g^-1^ | ^11^ |

**Table S2** Electrochemical performance of carbon materials in the anode of LIBs.

| Carbon material | Low current capacity | High current capacity | Reference |
| --- | --- | --- | --- |
| N-GHC | 50 mA g^-1^ 227 mAh g^-1^ | 1000 mA g^-1^ 104 mAh g^-1^ | This work |
| Natural graphite | 100 mA g^-1^ 150 mAh g^-1^ | 500 mA g^-1^ 125 mAh g^-1^ | ^12^ |
| Expanded graphite | 20 mA g^-1^ 284 mAh g^-1^ | 200 mA g^-1^ 91 mAh g^-1^ | ^13^ |
| Ion-catalyzed hard carbon | 100 mA g^-1^ 155 mAh g^-1^ | 1000 mA g^-1^ 100 mAh g^-1^ | ^14^ |
| Hard carbon nanoparticles | 25 mA g^-1^ 275 mAh g^-1^ | 2500 mA g^-1^ 45 mAh g^-1^ | ^15^ |
| Carbon nanofibers | 40 mA g^-1^ 255 mAh g^-1^ | 2000 mA g^-1^ 85 mAh g^-1^ | ^16^ |
| Porous carbon nanofibers | 50 mA g^-1^ 280 mAh g^-1^ | 5000 mA g^-1^ 90 mAh g^-1^ | ^17^ |
| Defective graphene | 100 mA g^-1^ 248 mAh g^-1^ | 5000 mA g^-1^ 154 mAh g^-1^ | ^18^ |
| Graphene-hard carbon | 20 mA g^-1^ 245 mAh g^-1^ | - | ^19^ |
| N-porous carbon fibers | 50 mA g^-1^ 296 mAh g^-1^ | 5000 mA g^-1^ 101 mAh g^-1^ | ^20^ |
| N-carbon nanofibers | 20 mA g^-1^ 150 mAh g^-1^ | 5000 mA g^-1^ 100 mAh g^-1^ | ^21^ |
| N-carbon nanosheets | 50 mA g^-1^ 350 mAh g^-1^ | 5000 mA g^-1^ 89 mAh g^-1^ | ^22^ |

**References**

1 Wang, G., Shen, X., Yao, J. & Park, J. Graphene nanosheets for enhanced lithium storage in lithium ion batteries. *Carbon* **47**, 2049-2053 (2009).

2 Yoo, E. J. *et al.* Large Reversible Li Storage of Graphene Nanosheet Families for Use in Rechargeable Lithium Ion Batteries. *Nano Lett.* **8**, 2277-2282 (2008).

3 Fan, Z. J. *et al.* Nanographene-constructed carbon nanofibers grown on graphene sheets by chemical vapor deposition: high-performance anode materials for lithium ion batteries. *Acs Nano* **5**, 2787-2794 (2011).

4 Wu, Z. S., Ren, W., Xu, L., Li, F. & Cheng, H. M. Doped graphene sheets as anode materials with superhigh rate and large capacity for lithium ion batteries. *Acs Nano* **5**, 5463 (2011).

5 Pol, V. G. & Thackeray, M. M. Spherical carbon particles and carbon nanotubes prepared by autogenic reactions : evaluation as anodes in lithium electrochemical cells. *Energy Env. Sci.* **4**, 1904-1912 (2011).

6 Guo, B. *et al.* Soft-templated mesoporous carbon-carbon nanotube composites for high performance lithium-ion batteries. *Adv. Mater.* **23**, 4661-4666 (2011).

7 Chen, L. *et al.* Porous Graphitic Carbon Nanosheets as a High-Rate Anode Material for Lithium-Ion Batteries. *Acs Appl. Mater. Interfaces* **5**, 9537-9545 (2013).

8 Chen, Y., Lu, Z., Zhou, L., Mai, Y. W. & Huang, H. Triple-coaxial electrospun amorphous carbon nanotubes with hollow graphitic carbon nanospheres for high-performance Li ion batteries. *Energy Env. Sci.* **5**, 7898-7902 (2012).

9 Li, W., Chen, M. & Wang, C. Spherical hard carbon prepared from potato starch using as anode material for Li-ion batteries. *Mater. Lett.* **65**, 3368-3370 (2011).

10 Wang, J., Liu, J. L., Wang, Y. G., Wang, C. X. & Xia, Y. Y. Pitch modified hard carbons as negative materials for lithium-ion batteries. *Electrochim. Acta* **74**, 1-7 (2012).

11 Han, F. D. *et al.* Template‐Free Synthesis of Interconnected Hollow Carbon Nanospheres for High‐Performance Anode Material in Lithium‐Ion Batteries. *Adv. Energy Mater.* **1**, 798-801 (2011).

12 Kim, H. *et al.* Sodium Storage Behavior in Natural Graphite using Ether-based Electrolyte Systems. *Adv. Funct. Mater.* **25**, 534-541 (2015).

13 Wen, Y. *et al.* Expanded graphite as superior anode for sodium-ion batteries. *Natu. Commun.* **5**, 4033 (2014).

14 Yu, Z.-L. *et al.* Ion-Catalyzed Synthesis of Microporous Hard Carbon Embedded with Expanded Nanographite for Enhanced Lithium/Sodium Storage. *J. Am. Chem. Soc.* **138**, 14915-14922 (2016).

15 Xiao, L. *et al.* Hard carbon nanoparticles as high-capacity, high-stability anodic materials for Na-ion batteries. *Nano Energy* **19**, 279-288 (2015).

16 Luo, W. *et al.* Carbon nanofibers derived from cellulose nanofibers as a long-life anode material for rechargeable sodium-ion batteries. *J. Mater. Chem. A* **1**, 10662-10666 (2013).

17 Li, W. H. *et al.* Free-standing and binder-free sodium-ion electrodes with ultralong cycle life and high rate performance based on porous carbon nanofibers. *Nanoscale* **6**, 693-698 (2014).

18 Pramudita, J. C. *et al.* Mechanisms of Sodium Insertion/Extraction on the Surface of Defective Graphenes. *Acs Appl. Mater. Interfaces* **9**, 431-438, (2017).

19 Luo, W. *et al.* Low-surface-area hard carbon anode for Na-ion batteries via graphene oxide as a dehydration agent. *ACS Appl. Mater. Interfaces* **7**, 2626-2631 (2015).

20 Fu, L. *et al.* Nitrogen doped porous carbon fibres as anode materials for sodium ion batteries with excellent rate performance. *Nanoscale* **6**, 1384-1389 (2014).

21 Wang, Z. *et al.* Functionalized N-doped interconnected carbon nanofibers as an anode material for sodium-ion storage with excellent performance. *Carbon* **55**, 328-334 (2013).

22 Wang, H. g. *et al.* Nitrogen‐Doped Porous Carbon Nanosheets as Low‐Cost, High‐Performance Anode Material for Sodium‐Ion Batteries. *ChemSusChem* **6**, 56-60 (2013).
